# Five stories: international scientists in China

**DOI:** 10.1093/nsr/nwac218

**Published:** 2022-11-17

**Authors:** Weijie Zhao

**Affiliations:** NSR news editor based in Beijing

## Abstract

A large number of Chinese scientists are working overseas, especially in the USA and Europe, where scientific communities are highly internationalized and diverse. In contrast, international scientists working in China are fewer, naturally generating some curiosity about them—why do they come to China, and how are their experiences in China? In this article, we introduce five such scientists and their stories.

## Follow the Mesozoic birds

**Jingmai O’Connor** took the position of the Associate Curator of Fossil Reptiles at the Chicago Field Museum of Natural History in 2020. Prior to this appointment, she had worked for over 10 years in China.

O’Connor's mother was born in China. She went to the USA in the 1970s and became a geochemist there. Influenced by her mother, O’Connor also became interested in rocks and fossils, and received her PhD in paleontology from the University of Southern California in 2009, with a thesis on the evolution of birds. ‘I work on Mesozoic birds. By the number of relevant specimens, 99% are found and housed in China. Thus, for me, going to China for my PhD to work with the renowned expert Zhonghe Zhou was a “no-brainer”.’ When she became Dr O’Connor, ‘I first intended to do postdocs at several institutes around the world but in the end, I could never bring myself to leave Beijing and the IVPP (Institute of Vertebrate Paleontology and Paleoanthropology)’. Thus, she stayed at the IVPP of Chinese Academy of Sciences (CAS), first as a postdoctoral researcher, then as an associated professor and finally as a professor.

O’Connor explores the evolution of flight in the Dinosauria, the dinosaur–bird transitional species, and the biology of stem-avians. She has published more than 130 papers and described 45 new species and unusual fossilized soft tissues in Cretaceous birds including lungs and traces of the ovary. She has conducted field work in China, Mongolia, Romania, Canada and South Africa, and now maintains an active field program in the USA. In 2019, she was awarded the Paleontogical Society's Schuchert Award, which honors a paleontologist under the age of 40 who demonstrates excellence and promise.

‘The decision to leave [the IVPP] was a hard one but ultimately, I was unable to get a green card which made starting a family in China unaffordable. I am still affiliated with the IVPP as an adjunct member, still training students there, and collaborating with colleagues,’ she said. Talking about the differences of conducting research in China and in the USA, O’Connor mentioned that in China, working under Prof. Zhonghe Zhou, the IVPP director, she never worried about funding and that ‘allowed us to pursue our research in whatever direction the specimens took us’. But back in the USA, she has to spend an enormous amount of time trying to get funding. She is also spending more time in science outreach now, as ‘the US is facing many crises related to science denialism’.

‘I dearly miss the Chinese subways which are affordable, clean, and on time,’ O’Connor said. The cultures and lifestyles in China and the USA are rather different, and it may take her some time to adapt to her new life. At the end of the interview, O’Connor told *NSR*: ‘I am grateful for the time I lived in China, the friends I made and the colleagues I will continue to work with. China really is a wonderful place to be a scientist.’

**Figure 1:**
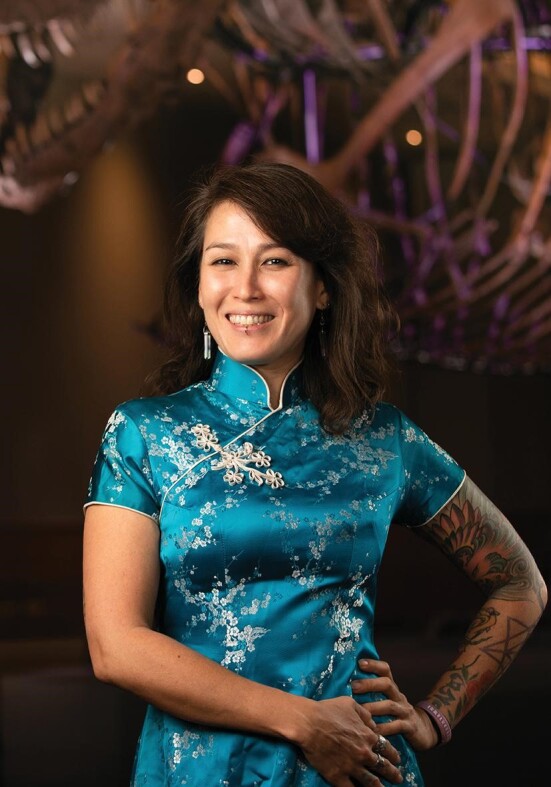
Prof. Jingmai O’Connor worked in China for >10 years and is now the Associate Curator of Fossil Reptiles at the Chicago Field Museum of Natural History (*Courtesy of Prof. O’Connor*).

O’Connor moved back to the USA but another oversea scientist, **Alida Bailleul**, is still working in the IVPP. Interestingly, O’Connor is the one who recruited Bailleul to China. Bailleul was a postdoctoral researcher at the University of Missouri in 2017. ‘I went to a conference on the east coast of the USA. There, I met Professor O’Connor. After hearing my talk, she talked to me about the possibility of applying for a fellowship to work with her and Prof. Zhonghe Zhou in Beijing,’ Bailleul recalled. She hesitated about moving to a new country where she could not speak the language, but considering that the IVPP is one of the top research institutes in the world for paleontology, she dived into this new adventure. She arrived in China in 2018 for a 2-year postdoctoral program and, in 2020, she became an Associate Professor of IVPP.

Bailleul's parents emigrated from France to the island of Tahiti in the mid-1980s and she was born there a few years later. She spent all her childhood climbing trees, jumping in rivers or swimming in the sea in this tropical paradise. She found the natural world fascinating and decided to become a paleontologist when she was in high school. Thus, she moved to France, obtained an undergraduate degree in biology and a master's degree in paleontology. After that, she moved to the USA, got a PhD degree in paleontology at Montana State University and worked at the University of Missouri as a postdoctoral researcher for a few years before she joined the IVPP in Beijing. She said: ‘I am very thankful in general for all of the opportunities that France, the US and China have given me during my career. China and IVPP have definitely played a major role in my scientific career, and I am very lucky to be able to work in this world-renown institute.’

In the IVPP, Bailleul managed to help set up an entire histochemistry laboratory to analyse the fossilized tissues of ancient birds and dinosaurs, mostly birds from the northeast of China in Liaoning Province. Birds and dinosaur fossils that are >100 million years old can still preserve remnants of their original soft tissues, such as feathers, skin, eyes, lungs or ovaries. Many fossils even preserve cells, cell nuclei and some original biomolecules. Bailleul's interest is to analyse these ancient tissues, extract the cells, figure out whether ancient cell nuclei are preserved and try

to test whether or not they may still retain any ancient DNA—not for cloning the ancient animals, but to understand the evolution of their genomes on a macroevolutionary scale. In 2020, Bailleul and colleagues reported evidence of proteins, chromosomes and chemical markers of DNA in exceptionally preserved dinosaur cartilage in *NSR*. ‘This type of research is controversial but truly fascinating to me,’ Bailleul said. ‘I did not expect so much success but my hard work and the support of my colleagues and supervisors have blessed me.’

In 2021, Bailleul was selected with a few other international scientists to meet the prime minister of China, Li Keqiang.

**Figure 2:**
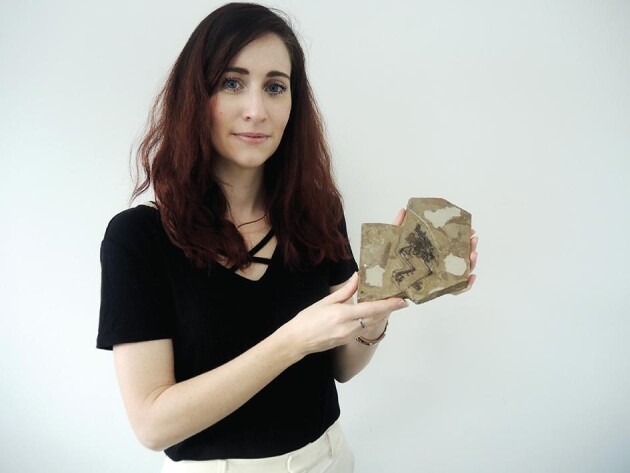
Alida Bailleul is an associate professor of the CAS Institute of Vertebrate Paleontology and Paleoanthropology (*Courtesy of Prof. Bailleul*).

## Learn about the ocean—on the other side of the PacIFIC

Similar to Alida Bailleul, **Brandon Bethel** was also born on a small island—in his case, an island of The Bahamas, located near the west coast of the Atlantic Ocean. In 2011, 19-year-old Bethel got an opportunity to come to China and learn the Chinese language at the Nanjing University of Information Science and Technology (NUIST) through the Confucius Institute at the then College of Bahamas (now University of The Bahamas). One year later, he became an undergraduate student of NUIST and, in 2022, he was awarded a PhD degree in marine meteorology at NUIST.

‘Like any other Bahamian, my life was closely intertwined with the ocean. Rather ironically, however, it was only after I came to China that I began to gain a deeper understanding of the ocean,’ Bethel said. His PhD program focused on physical oceanography, ocean numerical modeling, observations and artificial intelligence methods in oceanography. During the hardest months of the COVID outbreak in 2020, Bethel and his schoolmates were asked to stay in their dormitory rooms for most of the time. ‘I took the opportunity to identify where I had gone wrong with my work and fixed it,’ he recalled. ‘Failure is rarely pleasurable, but it is an excellent teacher. I eventually gained a mastery over my ocean wave model and even managed to write, submit, and publish a few papers in the first year of the pandemic.’

Bethel is currently in his home country and plans to return to China for postdoctoral studies. His home country, The Bahamas, is an archipelagic country with a small population of ∼400 000. ‘Doing research in China means that one has access to advanced high-performance computers, instruments, and the all-important finances, but this is not currently the case for academia in The Bahamas,’ Bethel said. He wants to take home the Chinese research culture and see what he can do to promote the nascent oceanographic community and to quicken the pace of economic growth of the Bahamas through harnessing the blue economy.

During the 11 years of studying in China, Bethel mastered the Chinese language and travelled extensively within China, ranging from the snow-covered Harbin in the north to the tropical Hainan Island in the south. ‘I fell in love with the culture,’ Bethel said. ‘The Bahamas has a special and close relationship with China and should encourage its citizens to travel there to learn and study as I was given a chance to.’

**Figure 3:**
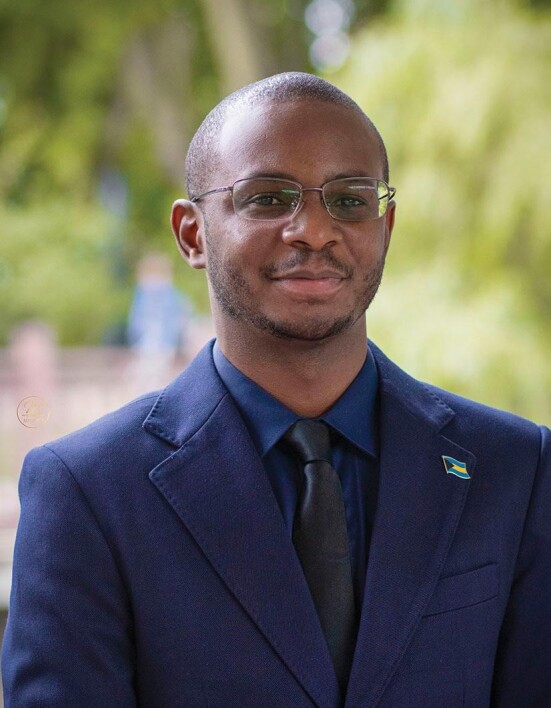
Dr. Brandon Bethel got his PhD degree in marine meteorology at the Nanjing University of Information Science and Technology (*Courtesy of Dr. Bethel*).

## Build a new lab in China

In 2018, **Alexey Kavokin** became the chair professor and director of the International Center for Polaritonics of the Westlake University—a newly established university located in Hangzhou, China. Westlake University is the first private non-profit research-oriented university in China and aims to become a world-renowned institution of higher education within 10–20 years. ‘I have been a Chair Professor in UK, head of two research labs in Russia and used to be a Research Director at the National Council for Research in Italy,’ Kavokin said. ‘I had to give up all these positions except the British one, which I still keep on a 25% basis.’

Kavokin was born in the former Soviet Union and got his PhD degree in physical and mathematical sciences in Russia. After that, he did postdoctoral research in Italy and become a professor in France, the UK and then China. Kavokin is a pioneer in the field of polaritonics—the physics of strongly coupled light–matter systems. His first experience with China was at the International Conference on the Physics of Light-Matter Coupling in Nanostructures, which was held in Hangzhou in 2012. ‘It was a very clear demonstration to the international scientific community of the high potential of physics in China,’ he recalled. ‘Since then, almost every year I came to Fudan University in Shanghai to give lectures.’

Then, in 2017, the Westlake University was founded and it began to recruit world-leading scientists to work on the highest-priority projects. ‘I estimated this as a high-risk-high-gain opportunity that might enable me to build a world best research center in my area,’ Kavokin said. ‘I feel very proud that I have won the competition and became one of the first Chair Professors of the Westlake University.’

The university offered Kavokin a very generous start-up fund and he has managed to build a new center in China. However, the pandemic made his transfer to China extremely challenging. ‘Two years under COVID was the most challenging period of my professional life,’ Kavokin said. ‘I cannot live and work without travelling between China and Europe. My four children and the most part of my research collaborations are located in Europe. I do need to travel a lot. Now it is nearly impossible.’

In spite of these difficulties, Kavokin is still confident that the difficult years will come to an end and is still very keen on bringing forward his research projects in China: ‘I am very proud of what we have achieved building in Hangzhou a world-leading research center, publishing many high-quality papers, and securing prestigious international awards such as the Quantum Device Award. We are training a lot of talented Chinese and international students. We are doing good science on a scale that would not be possible in Europe.’

**Figure 4:**
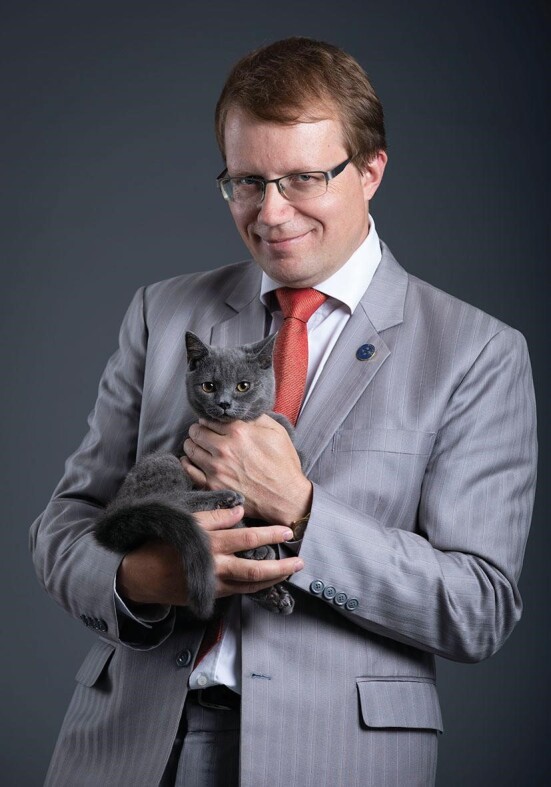
Prof. Alexey Kavokin is the chair professor and director of the International Center for Polaritonics of the Westlake University (*Courtesy of Prof. Kavokin*).

Kavokin has managed to set up his new center in China, while **Goran Angelovski** is still on his way to achieving that goal. At the beginning of 2020, the then director of the Max Planck Institute for Biological Cybernetics, Nikos K. Logothetis, decided to move to Shanghai to be the co-director of the newly established International Center for Primate Brain Research (ICPBR) of the CAS Center for Excellence for Brain Science and Intelligence Technology (CEBSIT), Institute of Neuroscience in Shanghai, and Angelovski is one of the principal investigators who moved together with Logothetis from Germany to China.

Angelovski got his bachelor’s (Hons) degree in his home country of Serbia and his PhD degree in Germany, majoring in medicinal chemistry and organic synthesis, respectively. ‘After obtaining my PhD title, my desire was to return to the research related to medicinal chemistry, so I looked for the appropriate labs to perform my postdoctoral studies,’ he recalled. ‘I came across the vacancy for a chemist at the Max Planck Institute for Biological Cybernetics, directed by Prof. Nikos K. Logothetis, for a researcher who could develop new type of imaging probes, called smart magnetic resonance imaging (MRI) agents.’ Angelovski took that opportunity and stepped into the new field of MRI and neuroscience.

‘The research environment consisting of biologists, physicists, mathematicians and engineers was indeed very stimulating, as we all had the same goal: better understanding of the brain function,’ Angelovski said. In the Max Planck Institute, he enjoyed full freedom in research and great trust by the department head. ‘From almost having no proper chemistry lab, we established a respectable research group that became recognizable among the peers.’ They designed, prepared and tested a number of new smart probes that can be effective in both basic research and clinical applications, and currently possess the largest own-developed library of calcium-sensitive MRI probes that are reported in the literature.

At that point, a chance to continue his research in China emerged for Angelovski. In the past couple of decades, Chinese scientists have made much progress in the development of biocompatible nanomaterials that can be used as carriers of MRI probes. That is one of the factors that attracted Angelovski to accept the opportunity and challenge to move to China. ‘The decision involved also important private aspects,’ he said. ‘Nevertheless with the great understanding and support of the entire family, we recently moved to Shanghai to continue our work or school education.’

However, even with the strong support of CEBSIT and the Shanghai government, Angelovski has not yet fully constructed his new lab in China, largely owing to the Covid pandemic. ‘Unfortunately, this “special” period has not finished yet, still causing many delays in the establishment of my lab and the entire center,’ he said. ‘I am currently mainly using the time to try to recruit new lab members, plan new projects, also to summarize and publish the results obtained at the former institute.’ Looking forward, Angelovski wishes that the pandemic will come to an end as soon as possible, the construction of his lab will be completed soon and the ICPBR will soon grow into a world-leading center for primate brain research as planned.

**Figure 5:**
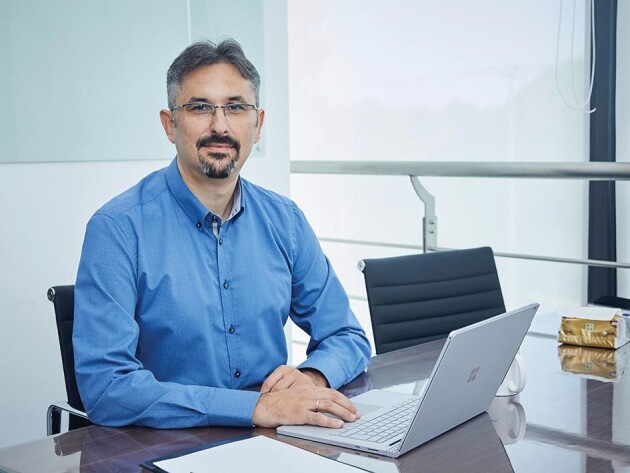
Prof. Goran Angelovski is the principal investigator of the Laboratory of Molecular and Cellular Neuroimaging, CEBSIT, CAS (*Courtesy of Prof. Angelovski*).

International collaboration and communication are important driving forces for future progress in science and technology. The Chinese scientific community has shown enthusiasm and readiness for welcoming international scientists at various stages of their career and of diverse academic backgrounds to come to China and participate in a wide range of research topics.

## Notes

Weijie Zhao is an NSR news editor based in Beijing

